# Challenges experienced by South African families caring for state patients on leave of absence

**DOI:** 10.4102/sajpsychiatry.v26i0.1453

**Published:** 2020-08-25

**Authors:** Nchaesa G. Mothwa, Miriam M. Moagi, Anna E. van der Wath

**Affiliations:** 1 Department of Nursing Science, Faculty of Health Sciences, University of Pretoria, Pretoria, South Africa

**Keywords:** burden of care, family, forensic patients, mental health, psychiatry, qualitative inquiry

## Abstract

**Background:**

Families of state patients experience challenges related to the patient’s mental illness and history of criminal behaviour. Family members who act as guardians when patients are on leave of absence take responsibility for the patient’s basic needs, activities of daily living and treatment regimen. They need to safeguard the patient from potential self-harm and harming others. Few studies have explored the burden these family members experience.

**Aim:**

The aim of this study was to explore and describe the challenges experienced by families caring for mental state patients who are on leave of absence.

**Setting:**

An urban area in South Africa.

**Methods:**

A qualitative approach was applied to answer the research question, ‘what are the challenges experienced by families caring for mental state patients on leave of absence?’ A purposive sample of nine participants who were caring for state patients on leave of absence was selected. Individual in-depth interviews were used to collect data. Data were analysed using thematic analysis. Ethical considerations and trustworthiness guided the study.

**Results:**

Three themes illustrate the challenges experienced by family members, namely, challenges related to state patient’s behaviour, emotional challenges and social challenges. A fourth theme focuses on the ways families used to cope with these challenges.

**Conclusion:**

Mental healthcare professionals may use the results of this study to design therapeutic interventions for family members of state patients who focus on empathetic understanding and the mobilisation of effective coping skills and social support.

## Introduction

The *South African Criminal Procedure Act*, No. 51 of 1977,^1^ makes provision for psychiatric forensic assessment of an accused person. If the accused suffers from a mental illness or intellectual disability that affects his or her competence to stand trial (Section 77) and/or criminal responsibility (Section 78), the court may order admission as a state patient (SP) for mental healthcare, treatment and rehabilitation according to Section 42 of the *Mental Health Care Act*, No. 17 of 2002.^2^ State patients are referred to as forensic patients in the international literature.

State patients are monitored for their potential risk to the community^3^ and, when stable, are considered for conditional or unconditional discharge, or reclassification as involuntary mental healthcare users. The review of SPs is performed 6 months after the initial contact and then 12 monthly thereafter.^2^ To facilitate community integration, SPs are granted leave of absence, where their behaviour is monitored regularly by clinicians.^4^ According to Section 45 of the *Mental Health Care Act*, No. 17 of 2002,^2^ the notice of leave of absence must state the terms and conditions the SP must comply with, for example, the patient must refrain from substances, stay in the care of the identified guardian and comply with treatment. Leave periods are extended if there are no adverse incidents and the patient remains stable, or can be cancelled if the patient does not comply with the conditions.^4^

The guardian (usually a relative identified by the SP and approved by the social worker) plays a crucial role when the patient re-enters society in terms of providing housing, monitoring of symptoms and treatment compliance and crisis intervention.^5^ The guardian, or his or her family, might have been recipients of the SPs’ offences.^5^ Whilst studies on the experiences of families and/or caregivers of mental healthcare users are readily available,^6,7,8^ only a few studies have explored the needs and burdens of caregivers of forensic patients.^9,10^ According to research conducted in Scotland,^9^ the burden of care affected caregivers’ physical and mental health.

In England, families of forensic patients experienced frustration and a lack of trust in the system of care, the staff members and the information received.^11^ Parents of forensic patients in Finland felt marginalised even though the patients relied heavily on them for support during re-entering the community.^12^ Caregivers in a rural area in South Africa experienced stress when the SPs presented with non-compliance to treatment and behaviour problems.^13^

The first author, a psychiatric nurse at a psychiatric hospital in an urban area in South Africa, realised that families of SPs who are on leave experience substantial challenges to fulfil their roles as caregivers and maintain the well-being of their families. Unable to cope with the situation, caregivers often return the patient to hospital and decline future guardianship. Inadequate social support contributes to institutionalisation and hinders SPs’ community re-integration. In order to meet caregivers’ needs, mental healthcare professionals need to understand their views and challenges,^11,12^ as well as the family strengths that enable them to cope.^10^ To the authors’ knowledge, no studies have been conducted in the study context on the challenges experienced by families of SPs who are on leave.

The aim of this study was to explore and describe the challenges experienced by families caring for SPs on leave of absence and to make recommendations to assist these families.

## Research methods and design

This study followed a qualitative descriptive approach grounded within a naturalistic paradigm.^14^ According to the naturalistic paradigm, there are multiple interpretations of reality, and the researcher aims to understand how participants construct reality within their context.^15^

### Setting

This study was conducted in a public psychiatric hospital that provides inpatient and outpatient mental health services to mental healthcare users with acute and long-term mental illnesses in an urban area in South Africa. State patients are admitted in a maximum security unit until they are stable enough to function in open units where they are prepared for leave of absence and discharge.

### Study population and sampling

The target population of this study included all families whose relatives are SPs and are on leave of absence. A non-probability purposive sampling was used to select participants.^15^ The nurses at the outpatient department assisted the researcher to identify eligible participants when they accompanied the SPs for follow-up. Potential participants were approached individually and introduced to the study. Those who met the following inclusion criteria were included in the study: (1) male or female family members (a parent, brother or sister) of those who had been SPs for at least 5 years, (2) who act as a caregiver for an SP, (3) over the age of 18 years, (4) able to communicate in English, Sepedi or IsiZulu and (5) able and willing to sign informed consent forms.

The sample consisted of five men and four women, of whom three were parents and six were either a brother or sister of an SP.

### Data collection

The first author collected the data during unstructured in-depth individual interviews, conducted in a private office at the hospital to ensure confidentiality. One open-ended question was asked: ‘what are the challenges that you experience when caring for your relative who is an SP whilst he or she is on leave of absence?’ The subsequent probing questions were guided by responses to the initial question. The audio-recorded interviews took an average time of 45 min and were transcribed and translated in English by the first author. A person proficient in Sepedi and IsiZulu checked the translations for correctness. A preliminary data analysis was conducted and interviews proceeded until data saturation was reached and themes were repeated and no new themes emerged.^15^

### Data analysis

Data were analysed using thematic analysis.^16^ All transcripts were read to get a sense of the whole. The transcripts were read again whilst searching for the challenges reflected in the participants’ statements. Ideas were written in the margin to describe the participants’ challenges. These ideas were abbreviated as codes. All transcripts were coded by writing the codes next to the appropriate segments of the text, using the most descriptive wording. Topics that related to each other were grouped together as themes. Themes were defined and named and quotes were identified to best demonstrate the theme.

### Rigour

To ensure trustworthiness of the findings, the criteria of credibility, transferability, dependability, confirmability and authenticity were used.^15^ The first author bracketed his own assumptions about the topic before he conducted the interviews. He recorded reflective notes about his own experiences after each interview to ensure credibility and dependability. An experienced coder and the first author analysed the data independently and reached consensus on the themes. A description of the setting and methods was provided to enhance confirmability and transferability. To ensure authenticity, the findings were supported by verbatim quotations.

### Ethical consideration

Ethical approval to conduct the study was obtained from the Faculty of Health Sciences Research Ethics Committee, University of Pretoria (reference number: 208/2017) and the management of Weskoppies Hospital, Pretoria, South Africa. The participants signed informed consent forms to ensure voluntarily participation and confidentiality was maintained as code numbers were assigned to transcribed interviews.

## Results

The interviews revealed four themes. Three themes illustrate the challenges experienced by families, namely, challenges related to SPs’ behaviour, emotional challenges and social challenges. The fourth theme describes the ways families used to cope with these challenges. The participant’s number is indicated in brackets after each verbatim quotation.

### Challenges related to state patients’ behaviour

Families struggled to come to terms with the crimes committed by the SPs. They found it difficult to manage the SPs’ behaviour and encouraged them to participate in activities of daily living (self-care, household responsibilities, social activities and adherence to treatment).

Some participants struggled to accept that the SP actually committed an offence, as illustrated in the following quotation:

‘The police said the incident [rape of a minor] happened in the street during the day and in full view of people but none have come up to say they saw the incident taking place.’ (P1, Male, Employed, < 40 years old)

The use of denial as a way of coping with the crime the SP committed is illustrated in the following quotation:

‘I was not convinced because when he grew up he was not a type of person to be involved in fights. He actually grew up as a so-called “coward.” We were very surprised because he has never gotten involved in naughty or illegal matters when he grew up. He has been sweet … I was really not convinced that he did it.’ (P4, Female, Unemployed, > 50 years old)

With regard to the SPs’ behaviour, one participant explained how they had to call the police on more than one occasion to take the patient back to hospital:

‘And in that process he will verbally abuse our mother, swearing and calling her names. At that time because of his aggression, we would always call the police who will help us take him back to the hospital.’ (P8, Male, Employed, > 50 years old)

Participants struggled to get the SPs to take responsibility for activities of daily living, for example:

‘He would wake up in the morning and not do anything let alone brushing his teeth. When reminded about that, he wouldn’t fight or get angry, he will just promise that he will do it later. Then he will sit and not say anything to us.’ (P9, Male, Pensioner, > 50 years old)

Some SPs presented with low drive and some used manipulative behaviour in order to be exempted from household tasks. The next participant avoided arguments with her brother in order to protect his feelings:

‘At times he responds and he would say “I am at home to rest, we work too hard at the hospital and besides they told me to rest” or “I am too tired I can’t do what you ask me to do, I have no energy” … we do not argue with him, we leave him as he is because we don’t want him to feel that being at home is a burden for him.’ (P5, Female, Employed, 41-50 years old)

The next quotation illustrates how the family encouraged the SP to get involved in family and community activities:

‘We also sit with him talking and reminding each other about old issues that happened in our lives. That’s the support we are giving him. We also orientate him to be conscious of time and current affairs. When it is time to watch news on television we always call him and encourage him to sit with us.’ (P1, Male, Employed, < 40 years old)

One participant expressed his dissatisfaction with his brother’s reluctance to attend church, but to rather associate with friends who abuse substances:

‘Another challenge is him not attending church as it is the norm in our family. We are from a Christian background and every Sunday we attend a church service. If he attends, he will not sit for more than thirty minutes; he will leave the service … My worry is that he also visits his friends … during his first leave of absence, my brother was involved in drugs and that was because of the type of friends he has.’ (P8, Male, Employed, > 50 years old)

Participants experienced challenges to monitor the SPs’ adherence to treatment, as explained in the next quotation:

‘It was only in the beginning when I think he had not come to terms with the fact that he was mentally ill … then we needed to be vigilant and firm to him when it came to taking his medication.’ (P8, Male, Employed, > 50 years old)

Some of the participants had to beg the SPs to adhere to their treatment, a reluctance they ascribed to the side effects of the medication:

‘When it came to medication, we had to beg him to take it. He used to argue that the fact that he was at home meant that he was no longer sick and hence there was no need for him to take his medication … Sometimes we would think that he was the way he was because of the medication he was taking. We were told that it makes people sleepy and tired. He also used to complain about it.’ (P9, Male, Pensioner, > 50 years old)

Families struggled to accept that their relatives committed a crime. Caregiving entailed a daily struggle to ensure that SPs perform activities of daily living and adhere to their treatment.

### Emotional challenges

Some participants expressed fear that the SP might present with aggression, violence and substance abuse, or try to abscond, as exemplified in the following quotations:

‘… I used to think as to what I will do if he became aggressive. Even with his friends, I thought and hoped that he associate himself with good ones. Since there are different types of drugs around here, I also feared that he might start using them. We thought he would do things as a result of peer pressure.’ (P4, Female, Unemployed, > 50 years old)

‘We were scared that he might leave the house unnoticed as he sleeps in the back room … I am at home with him all the time and mostly it is only the two of us, I am afraid that he might attempt to do something to me. At first I used to be scared when bringing him to the clinic, not knowing what he would do on the way. Whether he would be aggressive or run away.’ (P3, Female, Employed, > 50 years old)

One participant expressed fear and concern that the SP might relapse and act aggressively towards other family members whilst he is not at home:

‘I’m afraid that what if he becomes ill again … he might become violent towards my wife and kids and I don’t know what I will do if that happens. I will not forgive myself for that … it puts a toll on me though I want to believe that nothing will happen, but once I think that at home is only my wife and kids, I go straight home.’ (P2, Male, Employed, 41-50 years old)

Another participant explained that the sadness and emotional pain affected her sleeping pattern:

‘It hurts and makes me sad to see a grown-up man like him being dependent on us … also puts a strain on me when thinking about it. Sometimes I have sleepless nights because of that.’ (P3, Female, Employed, > 50 years old)

The next participant was so angry that she ended all contact with the SP:

‘I stopped all contact with him that I never visited him in prison. I was so angry with him for dragging our family’s name in such matters. I didn’t think he deserved my support as I used to tell him when he was growing up that I would not want him to associate himself with bad friends and behaviours. He stayed at the hospital for a long time with no visit since I said I dissociated myself from him …’ (P4, Female, Unemployed, > 50 years old)

Another participant felt hopeless at the thought of the crime her brother had committed:

‘I was devastated, I felt hopeless and I felt as if I was losing my mind because my brother couldn’t have done what they say he did.’ (P5, Female, Employed, 41-50 years old)

The burden of care and the crime the SP committed affected families emotionally and left them with the feelings of fear, anger and sadness.

### Social challenges

Inadequate social support and social stigma made it difficult for families to cope with the SPs. One participant suspected that the community harboured negative feelings towards the SP, although they never openly expressed these feelings.

He felt that he could not blame them for their feelings:

‘The community did not like what my brother did after they heard about it. They however have never said anything to us even though we all knew that they are talking about it … I understand that sometimes it is not easy to show support for a person who did things that are not in line with the law. I don’t blame them at all.’ (P2, Male, Employed, 41-50 years old)

Another participant described the negative responses from the community as follows:

‘You know with neighbours, some of them were laughing at us.’ (P5, Female, Employed, 41-50 years old)

Disappointment with the lack of family and community support is illustrated by one participant:

‘We have not had any support. I announced this matter at my church years back then and no one has ever come to sympathize with me. Now it has been 2 years since I went to church … my family has always been on its own. There has never been any support from them. Even visiting him in hospital, it’s only myself and other children. Relatives have never bothered about him or us.’ (P3, Female, Employed, > 50 years old)

Families who experience rejection and negative responses from community and family members have to bear the burden of care on their own.

### Ways of coping with challenges

Families of SPs dealt with their challenges through spiritual coping, acceptance and forgiveness. Some participants managed to mobilise community, family and institutional support.

The quotation below illustrates the use of religious coping:

‘We prayed and put our cries to the Lord. After all where will we run to? We kept praying … I told myself that I will leave everything in God’s hands.’ (P5, Female, Employed, 41-50 years old)

Most families did not harbour any bad feelings towards the SPs despite being directly affected by the offence. They coped through acceptance and forgiveness, for example:

‘We have forgiven him for what he has done. As for the present we felt that we should move on and not dwell on the past events. Yes, it saddened us, but we felt that the best way to deal with our loss is to forgive him.’ (P9, Male, Pensioner, > 50 years old)

When the SPs showed acceptable and helpful behaviour, their families found it easier to trust them, for example:

‘… [*T*]here aren’t any fears towards him. My son is not aggressive by nature. He gets along well with all. His nieces adore him a lot … He is always well behaved and there has not been any day that he has been aggressive towards anyone in the house.’ (P7, Female, Unemployed, > 50 years old)

‘We send him everywhere where we normally wouldn’t have reached. He is so helpful we wish he could come home for good since we are old now. We trust him so much these days that even our old age pension money is withdrawn by him when on leave. And he has never tried to steal any money from us.’ (P9, Male, Pensioner, > 50 years old)

Two participants explained how they coped through sharing their experiences with community members who were willing to support them with the burden of care:

‘It’s my friends and neighbours as I have told them about him. They always come to visit us and try to find out about his well-being. At times whenever he has to come for follow-up at the clinic and I am lacking in finances, they would help us out.’ (P4, Female, Unemployed, > 50 years old)

‘It will be church members asking about our well-being and they always enquire about our children as we have informed them about our tragedy when it happened … telling us that they will put us in their prayers.’ (P9, Male, Pensioner, > 50 years old)

Another participant expressed appreciation towards family and community members for their unconditional assistance:

‘… [*I*]f it was not through the help of his elder brother whom I call anytime when he becomes sick, I don’t know where I would have been … his sister also helped a lot in some situations where I nearly bore the brunt of his aggression … The only person giving us support from the community is our neighbour. My son fought with him for no reason once when he was on leave. After it happened, I went to apologise to him for what my son did to him. He did not hold a grudge against us.’ (P3, Female, Employed, > 50 years old)

Institutional support helped the families to cope through providing information regarding the SPs’ treatment, for example:

‘I put my request to the social worker who [would] then facilitate his leave. Once the leave is granted, I come to fetch him from the ward. In the ward prior to our departure, he will be given the treatment and it will be explained to him as to how to drink it and how many times.’ (P8, Male, Employed, > 50 years old)

Some families were willing to accept and forgive the SPs, whilst others used social and spiritual support to cope with their challenges.

## Discussion

Families need to come to terms with the crimes the SPs committed, even if the crime was directed towards a family member. As expected, these families experienced emotional and social challenges. What is unique to this study is that some of the families also shared positive experiences and managed to cope effectively with the situation.

Similar to this study, parents of forensic patients in Finland described how the crime aroused feelings of guilt, anxiety and powerlessness. Although the parents realised that it was in most cases impossible to predict the crime, they still tried to understand the reasons for the crime and how it might have been avoided. The results showed no direct association between the serious nature of the crime and the parents’ emotional experiences.^12^

Psychotic disorders represented the most common diagnostic category (69%) in a South African forensic unit, with schizophrenia being the most common diagnosis (44%).^17^ Although the diagnosis of the SPs in this study was not known, some participants feared potential violent incidents. In a study conducted in the United Kingdom,^6^ over 50% of the caregivers experienced incidents of violence from their relatives with psychosis, of which nearly two-thirds were directed towards the caregiver. Incidents of violence were associated with caregivers’ expressed hostility towards patients, lower self-esteem and the use of emotion-focused coping. State patients on leave of absence in a rural area in South Africa^13^ presented with disruptive behaviour, substance abuse, aggression and social isolation. They defaulted treatment that resulted in relapse and psychotic symptoms.^13^ The participants in the current study were also concerned about the SPs’ reluctance to participate in social activities and adhere to treatment.

The participants in this study experienced fear as they anticipated the SPs’ relapse. They were saddened by the SPs’ conditions and dependence on them. The first author bore witness to the participants’ painful emotions.

Being an experienced psychiatric nurse, he, however, managed to control his own feelings and express empathy with the participants’ experiences. Burdensome feelings such as self-recriminations, mistrust, anxiety, bitterness and depression were reported by parents of forensic patients.^12^ Caregivers were especially frustrated with SPs’ refusal to comply with treatment.^13^ Families might have more empathy if they understand the experiences of forensic patients, such as a fear of being lost without the sense of security the institution provides.^18^

A systematic review^10^ of eight studies concluded that families of forensic patients experienced significant stress because of violence, dual stigmatisation and disintegration of family relationships. Studies that compared the stigma attached to mental illness in general and when it is linked to offending produced conflicting results. Brooker et al.^19^ found that respondents were much less sympathetic towards psychiatric patients who had committed a crime, whilst Mezey et al.^20^ found similar levels of stigma in the general adult and forensic patients. In the current study, participants experienced judgemental attitudes from community members. They ascribed the lack of support from community and family members to stigmatisation. Without social support, families feel lonely and abandoned.^13^

In contrast to the families in this study who used a variety of coping skills, most families in the South African study^13^ felt unable to cope with the stressful situation of taking an SP on leave. In the Finnish study,^12^ the parents coped through avoidance, distraction, help seeking and religious coping. Coping strategies relied to a certain extent on the availability and strength of social support. Positive social support helped to alleviate negative feelings, and supported the family in everyday matters.^12^ The findings are comparable with the emotion-focused coping mechanisms used by caregivers of people with mental illness, namely, tolerance, positive thinking and help seeking, whilst social support helped them to cope with the psychological distress.^7^ Similar to research findings in the same study context,^21^ families in this study indicated that the absence of high-risk behaviour such as substance abuse and aggression facilitates trust during leave that increases the likelihood of SPs being discharged.

## Limitations

All participants in this study were black people, which means that other race groups were not represented in the study. The participants’ willingness to support the SPs might have reflected a skewed perspective. It is also possible that the families that were willing to participate could have done so because they had positive experiences to share, as opposed to the families that were not willing to be interviewed.

## Conclusion

Families need support to facilitate the successful community integration of SPs. Firstly, family members need to find ways to deal with their emotional experiences that originate from the crime committed by the SP, the patient’s mental health status as well as potential stigmatisation. Secondly, they require skills to manage SP’s challenging behaviour. Whilst some families are capable of mobilising a wide range of coping skills, including access to social support, others rely on mental healthcare professionals for support. Mental healthcare professionals need to employ appropriate interventions to assist families, for example, family and individual therapy and community-based psychoeducational interventions to learn coping skills and combat stigma.^22^ Crisis intervention should be accessible when the families find themselves at a dead end.
